# Analysis of Factors Limiting Bacterial Growth in PDMS Mother Machine Devices

**DOI:** 10.3389/fmicb.2018.00871

**Published:** 2018-05-01

**Authors:** Da Yang, Anna D. Jennings, Evalynn Borrego, Scott T. Retterer, Jaan Männik

**Affiliations:** ^1^Department of Physics and Astronomy, The University of Tennessee, Knoxville, TN, United States; ^2^Department of Mechanical, Aerospace and Biomedical Engineering, University of Tennessee, Knoxville, TN, United States; ^3^Oak Ridge National Laboratory, Center for Nanophase Materials Sciences, Oak Ridge, TN, United States

**Keywords:** mother machine, nutrient shielding, mechanics of cell growth, peptidoglycan synthesis, cell wall, microfluidics

## Abstract

The microfluidic mother machine platform has attracted much interest for its potential in studies of bacterial physiology, cellular organization, and cell mechanics. Despite numerous experiments and development of dedicated analysis software, differences in bacterial growth and morphology in narrow mother machine channels compared to typical liquid media conditions have not been systematically characterized. Here we determine changes in *E. coli* growth rates and cell dimensions in different sized dead-end microfluidic channels using high resolution optical microscopy. We find that *E. coli* adapt to the confined channel environment by becoming narrower and longer compared to the same strain grown in liquid culture. Cell dimensions decrease as the channel length increases and width decreases. These changes are accompanied by increases in doubling times in agreement with the universal growth law. In channels 100 μm and longer, cell doublings can completely stop as a result of frictional forces that oppose cell elongation. Before complete cessation of elongation, mechanical stresses lead to substantial deformation of cells and changes in their morphology. Our work shows that mechanical forces rather than nutrient limitation are the main growth limiting factor for bacterial growth in long and narrow channels.

## Introduction

High resolution optical microscopy is the most widespread method to study bacterial cellular organization and physiology at the single cell level. Most early studies were carried out using fixed cells that were attached to microscope slides. However, both fixation and attachment of cells to slides alter subcellular organization and can lead to imaging artifacts. As a less invasive preparation method, thin agarose pads sandwiched between microscope cover slide and coverslip have been adopted by numerous groups (e.g., in recent reports, Bailey et al., 2014; Adiciptaningrum et al., 2015). Since thin layer of agarose is prone to drying, thicker layers of agarose in Petri dishes with coverslip bottoms can be used (Männik et al., 2017). The dishes and pads allow imaging live cells over several doublings (typically 4–5). Longer imaging is hampered because individual cells start to overlap. Moreover, cells in the interior of the colony experience a different growth environment than the cells at the periphery and consequently grow at different rates. It is unclear for how long exactly, if at all, steady-state growth conditions can be maintained on the pads.

For reproducible quantitative studies, steady-state cell growth is necessary. To be able to grow cells in steady conditions both the physical and chemical environment of cells needs to remain the same over time. Moreover, cells should not overlap as they grow. In practical terms this means that the colony size has to be kept fixed despite exponential growth in cell numbers over time. Different microfluidic platforms have been developed over the past decade to achieve these requirements (Hol and Dekker, 2014). The developed devices either trap cells in narrow channels comparable to bacterial cross-sectional diameter (Wang et al., 2010; Moffitt et al., 2012; Long et al., 2013) or hold them in shallow chambers where bacteria are confined to a single layer (Männik et al., 2012; Ullman et al., 2013). In the latter case bacteria grow packed side-by-side and quantitative analysis of individual cells is more complicated. In addition to providing a steady growth environment, microfluidics can also be used to administer different chemical (Baltekin et al., 2017; Kaiser et al., 2018) and physical stimuli (Yang et al., 2015) to the cells *in situ* while they are imaged under the microscope.

**Figure 1:**
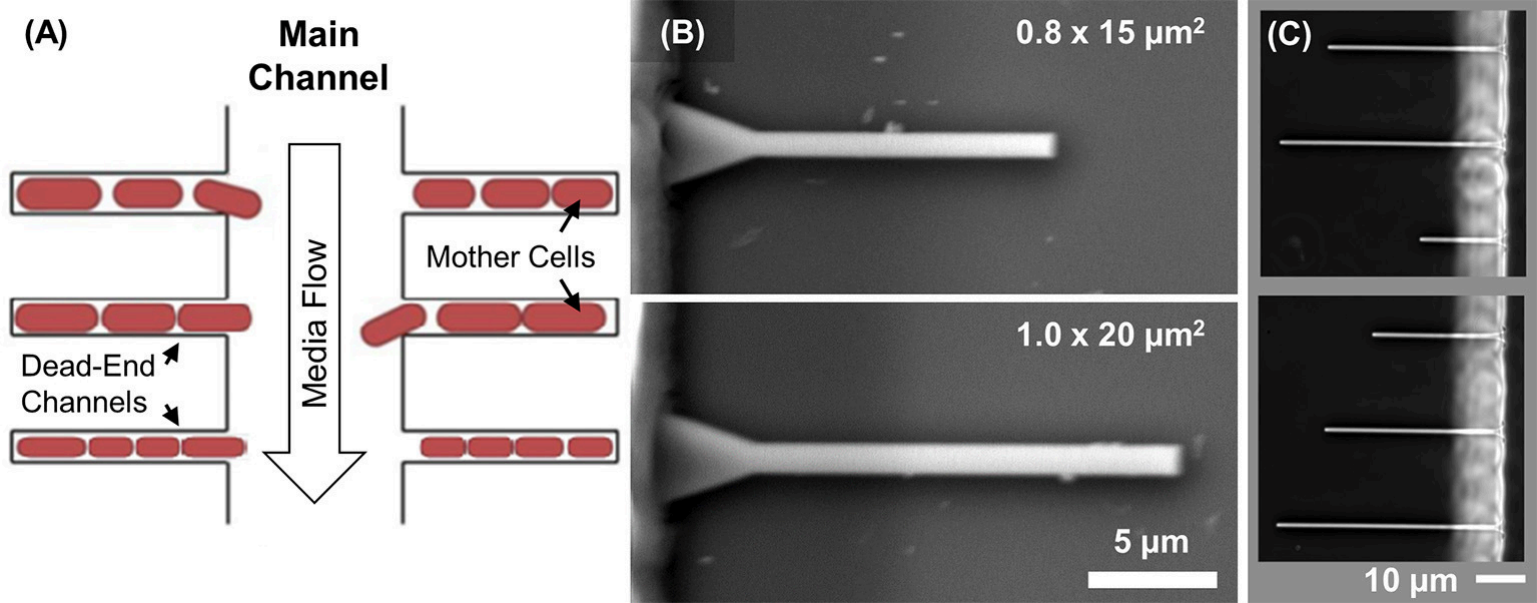
Design of microfluidic chip. **(A)** Schematics showing the mother machine channel layout. Cells grow in dead-end channels. Nutrients diffuse to cells from the main channel where a constant flow is maintained. This flow also removes metabolic waste products and flushes away extra cells. Growth of the mother cell at the end of the channel is studied as a function of channel width and length. **(B)** SEM images of the silicon mold showing patterns of two different size channels. **(C)** Channels form a completed PDMS device imaged using phase contrast microscopy. Channel lengths in the two images vary from 20 to 50 μm. There is a total of 150 channels of each length on a single chip.

Of these various designs the most wide-spread has been the so-called mother machine platform (Wang et al., 2010) where cells grow in short (10–25 μm long) dead-end channels (Figure 1A). The advantage of dead-end channels relative to channels where both ends are open is longer retention time of cells. Pressure fluctuations are more likely to drive cells out from the channels that have both ends open. In mother machine design, all the cells in the channels are clones of the mother cell that resides in the dead-end side of the channel. The size of the colony is maintained fixed in time because flow in the main channel flushes away extra cells that grow out from the dead-end channels. The same flow also maintains a constant media environment in the growth channels by replenishing nutrients and removing metabolic waste products. Both exchanges are thought to occur via diffusion (Wang et al., 2010). Diffusion may set a limit for nutrient availability for cells at the dead-end side of the channel. To increase diffusion rate a design with shallow reservoirs surrounding the dead-end channels has been implemented (Norman et al., 2013; Cabeen et al., 2017). These reservoirs allow diffusion of nutrients from the main channel but are shallow enough to prevent cells from populating them. Faster exchange of media can also be achieved by diverting some flow past the cells via small opening on the “dead-end” side of the channel. While allowing flow of medium, the opening needs to be made small enough to prevent cells from passing through. Such channels have been recently fabricated and tested (Baltekin et al., 2017; Jennings, 2017). However, the fabrication of these devices is rather challenging, especially for smaller size bacteria, such as *E. coli* growing in poor medium, because the opening in the dead-end side needs to be made no more than about 300 nm wide to prevent cells from squeezing through the deformable openings (Männik et al., 2009; Jennings, 2017).

Mother machine platform has been used to study cell aging (Wang et al., 2010), cell cycle control (Taheri-Araghi et al., 2015), and effects of mechanical forces on cell wall growth (Amir et al., 2014). The devices have been also used in studies of gene regulation (Norman et al., 2013; Cabeen et al., 2017; Kaiser et al., 2018) and antibiotic resistance (Baltekin et al., 2017). New open-source computational platforms have been specifically developed to segment and analyze cells in mother machine platform (Sachs et al., 2016; Kaiser et al., 2018). Despite such widespread interest, bacterial growth in narrow dead-end channels has not been systematically compared to their growth in typical liquid media conditions. Nor has it been determined what phenotypic differences appear in cells that grow in such microfluidic devices. Here, we analyze nutritional and mechanical growth limitations to clonal *E. coli* cultures in microfluidic dead-end channels of various widths and lengths (Figure 1A). We find that *E. coli* adapt to the confined channel environment by becoming significantly narrower and longer than the same bacteria in liquid cultures. While the aspect ratio is affected, the cell volume remains approximately the same for cells growing in short channels compared to those growing in the same media conditions in liquid cultures. By increasing the length of the channels, the growth speed and cell volume both decrease until cell growth completely stops in longer channels. We assign the complete cessation of growth to high levels of mechanical stress resulting from colony growth rather than from nutrient limitations. Interestingly, the stress in these 1D colonies can reach levels sufficient to deform cells and to cause them growing into irregular shapes.

## Materials and methods

### Microfluidic device design and fabrication

The fluidic circuitry in each chip consists of the main channel for media supply and waste product removal and 600 dead-end channels connected to the main channel (Figure 1A) following the typical mother machine layout (Wang et al., 2010). The designed length of the dead-end channels varies between 15 and 200 μm, and width from 0.6 to 1.0 μm. The fabrication of microfluidic devices is based on soft-lithography of polydimethylsiloxane (PDMS) elastomers (Weibel et al., 2007).

The channels in PDMS elastomers are created using 4″ silicon (Si) wafer molds. The fabrication of the Si molds follows the process described earlier (Yang et al., 2015). Briefly, the patterns of dead-end channels are defined by e-beam lithography using a JEOL JBX-9300FS electron beam lithography system (JEOL, Japan) with ZEP520A, a positive tone e-beam resist (ZEON Chemical, Japan). After e-beam writing and resist development, a 15 nm chromium layer is deposited. Subsequently, the e-beam resist layer is lifted off using sonication in an acetone bath. A 1.2 μm deep Si etch is carried out in an Oxford Plasmalab 100 inductively coupled plasma reactive ion etching system (Oxford Instruments, MA). The Cr layer acts as a mask for Si etching. The patterns for the larger flow channels are defined using photolithography of SU-8 2015 (MicroChem, MA). The reliefs that result from this step have a typical height of 20 μm. The molds are subsequently silanized in a desiccator using (tridecafluoro-1,1,2,2-tetrahydrooctyl)-1-trichlorosilane (UCT Specialties, CA) for at least 15 min.

PDMS elastomer (Sylgard 184 kit, by Dow Corning, MI) is cast on the mold in a 10:1 base/linker weight ratio. The PDMS is baked at 90°C for 20 min in a convection oven, and then left in the oven for at least two more hours as the oven cools down from 90°C. Individual patterns are cut out, and access holes to the main channels are punched using a biopsy needle. These pieces are subsequently bonded to coverslips. For bonding, #1.5 coverslips are cleaned in isopropyl alcohol (both from Thermo Fisher Scientific, NH) by sonication and then treated in O_2_ plasma at 200 mTorr for 70 s. The PDMS elastomer piece and glass coverslips are additionally treated in O_2_ plasma for 7s before bonding. After bonding, the chips are left at room temperature at least for 12 h before starting live cell measurements.

The channel heights and widths are measured from the Si mold. For the height measurement, a KLA-Tencor P-6 Stylus (KLA-Tencor Corporation, CA) profilometer is used. The heights of all dead-end channels in different microfluidic chips are within 1.15 ± 0.05 μm. The channels widths are measured using SEM and closely follow their design widths (less than 20 nm differences) (Figure 1B). The length of these channels, as measured from the optical images of completed chips (Figure 1C), also closely follow their design values.

### Bacterial strain and culturing

In all measurements, *E. coli* strain AJ5 is used. The strain is created from strain BW25113 (Datsenko and Wanner, 2000) by P1 transduction with lysate from strain FW1401 (Wu et al., 2015). The resulting strain carries a *tagRFP-T* sequence together with kanamycin resistance cassette replacing *leuB*. For an experiment, a colony from LB plate is grown overnight at 28°C with shaking in M9 minimal medium. The medium consists of M9 salts (Teknova, CA), supplemented with 2 mM magnesium sulfate (MgSO_4_), 0.5% (w/v) glucose (Sigma-Aldrich, MO), 0.2% casamino acids (ACROS Organics, NJ). Liquid media are supplemented with 25 μg/ml kanamycin (Sigma-Aldrich, MO) for selection.

#### Microfluidic chip experiment

Before inoculation of cells to a microfluidic chip, 0.1% (w/v) bovine serum albumin (BSA, Sigma-Aldrich, MO) is added to an overnight liquid culture (OD_600_ > 0.8). The cells are then concentrated 100 times in the same medium. About 2 μl of the concentrated culture is injected into the microfluidic device using a pipette and left at 28°C. After a satisfactory amount of dead-end channels are filled with at least one cell (requires a minimum of 20 min), tubing will be connected to the device, and the flow of fresh M9 medium is started. The medium that is used is the same as for the overnight culture, but with 0.1% BSA added to prevent cells from sticking to the main channel surfaces. The medium contains no antibiotics. The flow of this medium is maintained at 4.5 μl/min by an NE-1000 Syringe Pump (New Era Pump Systems, NY). The micro-cultures are grown at 28°C for at least 14 h before imaging is started.

#### Liquid culture measurements

To measure doubling times in liquid culture, the cells are grown in M9 medium overnight (OD_600_ > 0.8). The composition of the medium is the same as for the microfluidic chip measurements. The culture is then diluted at least 100× to fresh M9 medium. OD of the culture is measured at λ = 600 nm using a GENESYS 20 Visible Spectrophotometer (Thermo Fisher Scientific, NH) in 30 min intervals outside the incubator. The linear region of the log(OD) vs. *t* curve is used to determine the doubling time (Supplementary Figure 1).

To measure cell dimensions in liquid culture, cells are grown to mid-log phase (OD_600_ ~ 0.15), concentrated 50×, and then spread onto a 2% agarose pad with the same M9 media composition as in other measurements. The cells are imaged within 30 min after spreading to the pad.

### Microscopic imaging

A Nikon Ti-E inverted epifluorescence microscope (Nikon Instruments, Japan) with a 100X (NA = 1.45) oil immersion phase contrast objective (Nikon Instruments, Japan) is used for imaging the bacteria. Images are captured on an iXon DU-897 EMCCD camera (Andor Technology, UK) and recorded using NIS-Elements software (Nikon Instruments, Japan). Fluorophores are excited by a 200 W Hg lamp through an ND4 neutral density filter. A Chroma 41004 filtercube (Chroma Technology Corp., VT) is used when capturing fluorescent images. A motorized stage and a perfect focus system are utilized during time-lapse imaging. About 100 dead-end channels for each channel size is imaged in each measurement for 6-h period or longer (at least 5 doublings).

### Image analysis

Image analysis was carried out using Matlab (MathWorks, MA) scripts based on Matlab Image Analysis Toolbox, Signal Processing Toolbox and DipImage Toolbox. ImageJ was used to prepare individual images for the figures and the SI movies.

#### Cell length measurements

Both cell lengths and widths are measured from fluorescent images of the cytoplasmic tagRFP-T label. First, the raw fluorescent images are transformed to the second derivative images using the DipImage function *laplace_plus_dgg*, which computes the Laplacian and the second derivative in the gradient direction of an image (Verbeek and Vanvliet, 1994). Thresholding of the second derivative image based on zero crossing yields a binary image, which is then eroded and dilated to resolve individual cells and to fill them, respectively. Cell lengths are measured from these binary images. The measurement consists of finding the greatest Feret diameter for each cell using the *measure* function from DipImage.

#### Cell width measurements

Based on the binary cell masks from the previous step, the gray value center of mass coordinates and the coordinates of the cell long axes are determined. Next, a line perpendicular to the long axes of the cell that passes through the cell center is calculated. To improve the determination of the cell width, an additional set of lines are calculated, where the orientation relative to the previous line is varied in small angular steps. For each orientation, an intensity profile from the fluorescent image is determined, and the profile is fitted to a Gaussian. The Gaussian with the smallest width is then found among all the fits. Due to diffraction, variance of the Gaussian is about 0.04 μm smaller than the cytoplasmic diameter of the cell in our setup (Männik et al., 2009). An additional 0.04 μm is added to the cytoplasmic diameter to account for the width of the periplasmic space and outer membrane layer of the cell. The final, calculated width thus corresponds to the outer diameter of the cell.

#### Cell volume measurements

For the cell volume determination, we assume that every cell is a cylinder with two hemispherical caps. The volume, *V*_*c*_, based on cell length, *L*_*c*_, and width, *W*_*c*_, is:

(1) $$V_{c}=\;\frac{\pi }{6}{W_{c}}^{3}+\;\frac{\pi }{4}{W_{c}}^{2}(L_{c}-W_{c})$$

### Statistical analysis

Three replicate measurements of cells were performed for each channel size and for liquid cultures. The average cell length, width, volume and doubling time were calculated from the averages of these three measurements. The error bars associated with the above measurements are the standard errors which have been calculated from the averages of the three measurements. For universal growth law plot the error bars were calculated by propagating the random errors for volumes and doubling times. For statistical significance testing Welch's *t*-test was used. This test is more reliable than Student's *t*-test when sample sizes are different. To estimate correlations Pearson R was used.

## Results

### Dependence of mother cell growth on channel width

**Figure 2:**
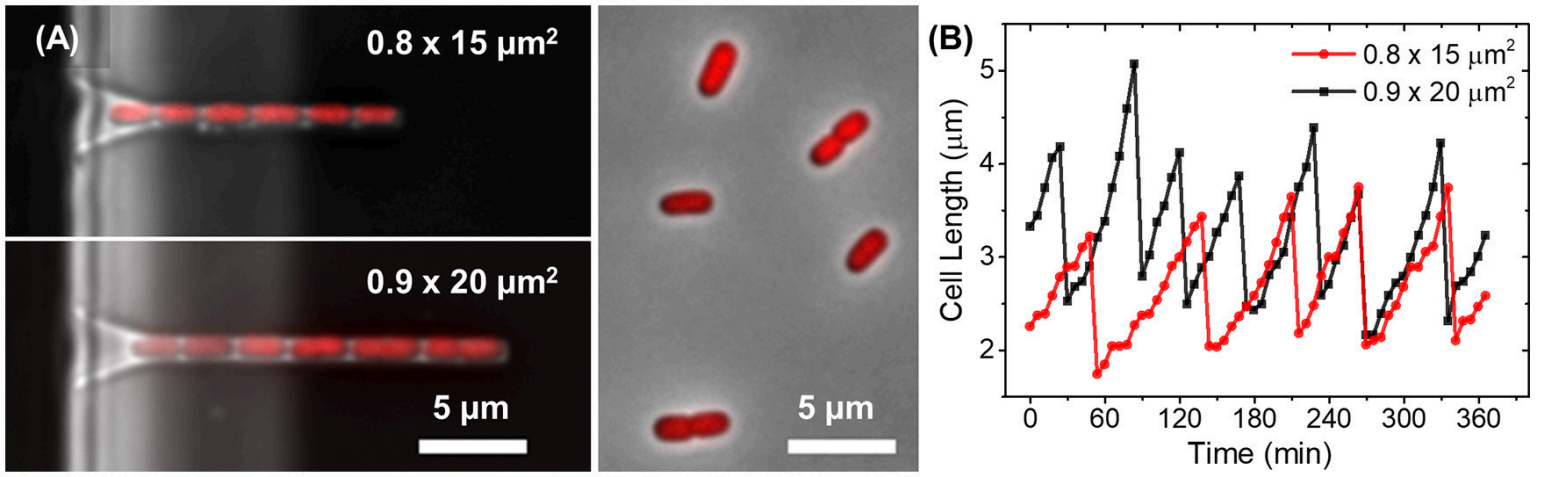
Bacterial growth in channels. **(A)** Composite of phase and fluorescent images of two different size channels filled tagRFP-T labeled *E. coli* (left images). For comparison, composite image of the same strain grown in liquid culture and imaged on agarose pad (right image). The difference in cell width in the two growth environments can be visualized. **(B)** Growth curves of mother cells from channels shown on **(A)**.

To investigate how the channel width affects the growth rate and cell dimensions we fabricated channels of 0.6, 0.7, 0.8, 0.9, 1.0, and 1.2 μm in width, 1.15 μm in height and of 15 and 20 μm in length on a single chip. The chosen channel widths were expected to be close to the cell diameter in these growth conditions. The majority of the 0.8, 0.9, and 1.0 μm wide channels had stable bacterial populations that filled the whole channel. Cells in these populations grew in single rows (Figure 2A, Supplementary Videos 1, 2). In the 1.2 μm wide channels, cells grew in two parallel rows and, as such, these channels were not suitable for analysis. On the other hand, 0.6 μm wide channels were too narrow to support stable colony growth. Although few cells loaded to these channels initially, they all moved out from the channels before imaging started. The same also occurred in the majority of the 0.7 μm wide channels (Supplementary Video 3). However, in some of the 0.7 μm wide channels (8 out of 200), stable populations were present and could be imaged. In both 0.6 and 0.7 μm wide channels the bacteria appeared to be wider than the channel causing deformations to channel walls (Supplementary Figure 2). Note that bacteria from overnight stationary cultures were loaded into channels. These cells are significantly narrower than log phase cells (Männik et al., 2009), and this made their entry to 0.6 and 0.7 μm wide channels possible. As cells started to grow in fresh medium in these channels, their diameter widened beyond the channel width. Higher deformability of open ends of channels may have provided a driving force that pushed the cells out from the channels.

Thus, of all the fabricated channels only those in the range 0.7–1.0 μm were suitable for studying growth of stable 1D bacterial cultures. Cells in these channels were imaged in three independent measurements that each lasted at least 6 h (Supplementary Videos 1, 2, Figure 2B). Here, we analyze, in detail, the growth of the mother cell, which is the cell at the dead-end side of the channel. All the cells in the channel are clones of this cell. We use the mother cell for analysis because it is the only cell that strictly grows in steady state conditions. The other cells in the channel move toward the channel entrance during their growth and, because of that movement, we expect both the composition of the growth medium and the mechanical stress that the cells experience to change. Also, to guarantee that mother cells in a channel of a given size grew in comparable conditions we required the channels to be completely full of cells (<1 μm empty space between cells) throughout the entire time-lapse imaging period.

**Figure 3:**
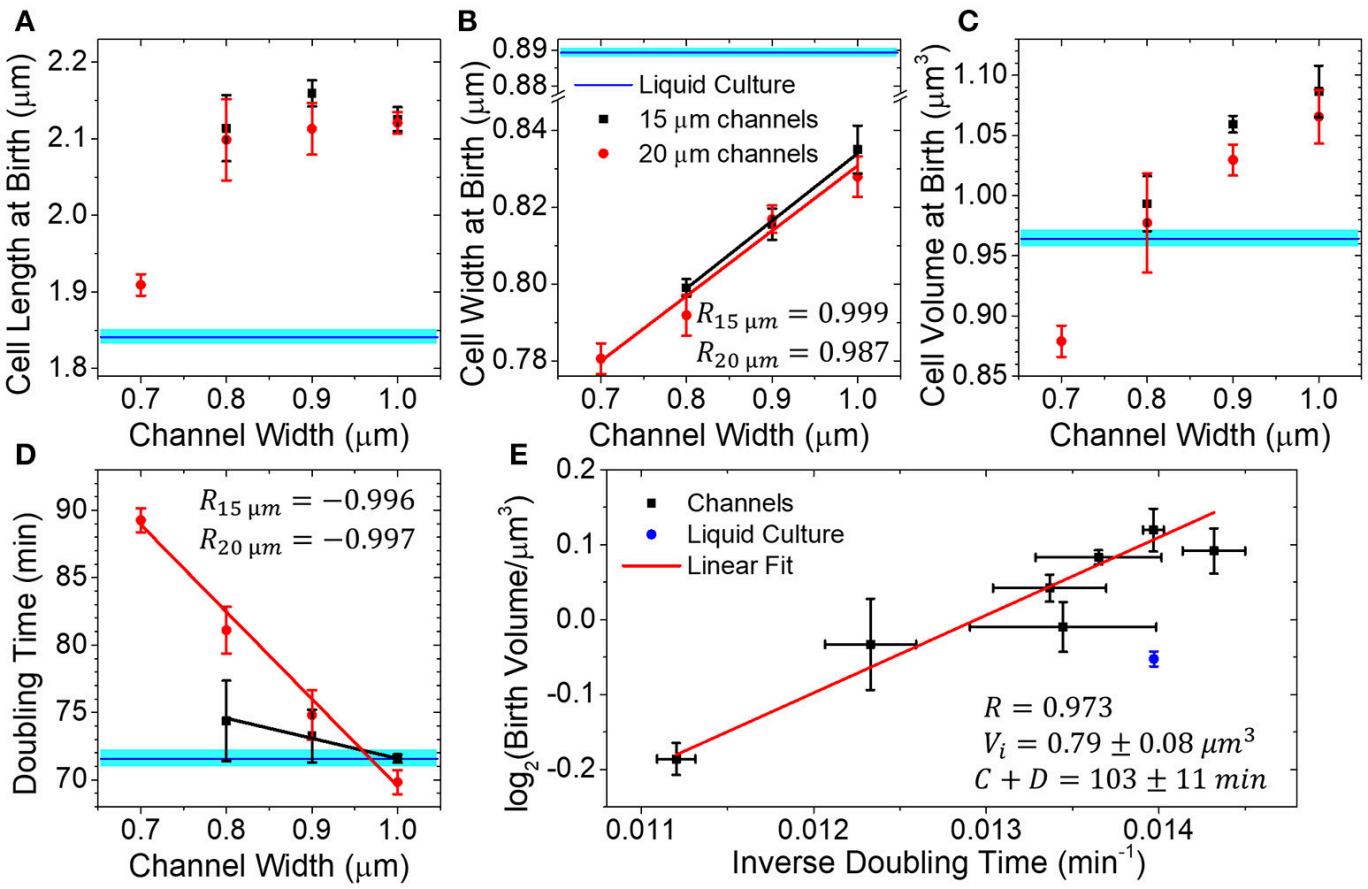
Dimensions of the mother cell and its doubling times in dead-end channels of different widths. **(A–D)** Mother cell length, width, volume and doubling time as a function of channel width in channels of 15 (red circles) and 20 μm (black squares) length. All measurements have been done at 28°C. Data points represent the average of three independent measurements. In each measurement, more than 300 cell births have been analyzed. Error bars are s.e.m. of the three independent measurements. Blue horizontal lines represent measurements of cells from liquid culture tubes. The width of these lines corresponds to s.e.m. All other solid lines are linear fits to the data. **(E)** Logarithm of birth volume, *V*_*b*_, as a function inverse of doubling time, $T_{d}^{-1}$. Solid line shows the fit of the data to the universal growth law, $V_{b}=\left(\frac{1}{2}\right)V_{i}•2^{\frac{C+D}{T_{d}}}$. In the universal growth law, *V*_*i*_ is cell volume at the initiation of replication, *C* the time taken to replicate DNA and *D* the time taken from the end of replication to cell division. Here *V*_*i*_ and *C*+*D* are treated as fitting parameters with best fit values $V_{i}=0.79\pm 0.08\;\mu m^{3}$ and *C*+*D* = 103±11 min. The errors are standard error.

We next compare birth length, width, volume and doubling time measurements of mother cells in differently sized channels to those from cells in liquid cultures. We found cell length (2.12 ± 0.05 μm) to be independent of channel width in the 0.8–1.0 μm range (Figure 3A). In 0.7 μm wide channels, where cells appeared to be in contact with channel walls, the cell lengths were significantly (9% difference, *p* < 0.02) smaller than those in other channels. Interestingly, cells were longer in all microfluidic channels than in the test-tube liquid cultures (1.84 μm; *p* = 0.19 for 0.7 μm wide channel, and *p* < 10^−16^ for all other channels). At the same time, the coefficients of variation of the cell length at birth distributions were comparable to the values in the liquid culture (all are about 15%) (Supplementary Figure 3). The coefficients of variation determined here are in agreement with previous measurements where values of 12–17% have been reported from agarose pad (Adiciptaningrum et al., 2015) and microfluidic measurements (Campos et al., 2014; Taheri-Araghi et al., 2015).

While cell lengths were longer in microfluidic channels, their widths were significantly smaller (Welch's *t*-test *p* < 10^−8^) (Figure 3B). Also, coefficients of variation of the cell width distribution were smaller than the ones in liquid culture (4.5%) and decreased as the channel width decreased from 4 to 3% (Supplementary Figure 3). In 0.7 μm wide channels, the cell width was wider than the undeformed channel diameter indicating that channel walls prevented cells from becoming wider. In 0.8 μm wide channels, some cells could still be squeezed by channel walls. In 0.9 and 1.0 μm wide channels, however, the cells were narrower than the channels and not squeezed by the channel walls. Irrespective of squeezing or no squeezing from channel walls, the cell width at birth increased linearly with the channel width (Pearson *R* = 0.999 in 15 μm long and 0.987 in 20 μm long channels). So, in wider channels, the decrease in cell diameter was not linked to a direct mechanical force to cells but must have reflected some form of adaptation of the cells to a confined channel environment.

As expected from the two above measurements, the average mother cell volume also increased with the channel width (Figure 3C). Comparison of microfluidic and the liquid culture cells showed that the differences in their lengths and widths, to some degree, compensated each other in cell volumes. In particular, for the 0.8 μm wide channels, the cell volumes matched those in liquid culture.

The doubling times also depended on channel width and increased as the channels become narrower (Figure 3D). The effect was pronounced for cells in 20 μm long channels (*R* = −0.997, slope = −65 min/μm), but was less significant in 15 μm channels (*R* = −0.996, slope = −15 min/μm). In the latter case, the doubling times appeared almost indistinguishable from cells grown in liquid cultures despite changes in cell shape. The observed increase in cell doubling times with the decrease of its dimensions is expected. According to the universal growth law, cell volumes at birth depend exponentially on growth rate, which is taken here as an inverse of doubling time (Schaechter et al., 1958; Willis and Huang, 2017). Such correlations are indeed evident in our data (Figure 3E). The universal growth law emerged from studies where cells grew unrestricted, and the growth rate was determined by the type of carbon source rather than by its abundance (Schaechter et al., 1958). In later studies, growth rate was also examined in growth limiting conditions and the increase in cell size and growth rate were found to be correlated in most but not all conditions (Shehata and Marr, 1971). Accordingly, we interpreted the exponential decrease in mother cell volume as a function of doubling time to result from nutrient limiting conditions in channels ends which would arise from nutrient shielding in the channel.

### Dependence of mother cell growth on channel length

To further investigate this hypothesis, we studied how the dimensions and doubling times of mother cell depended on the channel length. We expected that doubling times should increase and cell dimensions should decrease as the length of the channel increases. For these studies, we used microfluidic chips with a fixed channel width of 0.9 μm but varied channel length from about 20–50 μm (19, 29, 39, and 49 μm). We combined results from these measurements with the earlier ones in 15 and 20 μm long channels. Although the 15 and 20 μm long channels had the same designed width as the longer channels, the actual widths of longer channels appeared slightly smaller than 0.9 μm, which likely explains the differences in growth rates of the two data sets.

**Figure 4:**
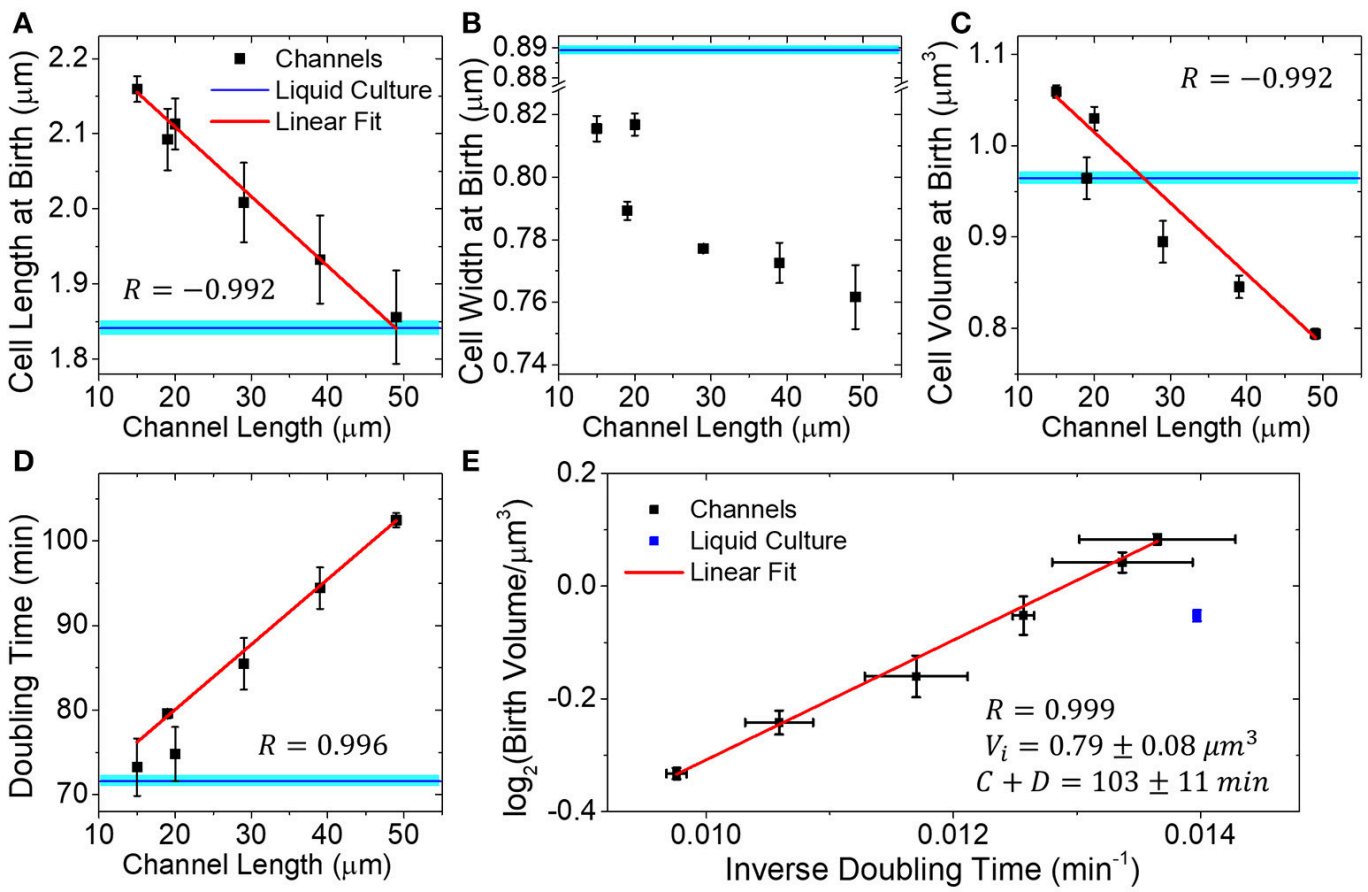
Dimensions and doubling times of mother cells in channels of different lengths. **(A–D)** Mother cell length, width, and volume at birth, and doubling time as a function of channel length. All channels are 0.9 μm wide. Each data point is an average of three independent measurements. In each measurement, more than 300 cell births have been analyzed. Error bars are s.e.m. of the three independent measurements. Blue horizontal lines correspond to measurements of these quantities from liquid culture cells. Solid lines are linear fits to data. **(E)** Logarithm of birth volume, *V*_*b*_, as a function of inverse doubling time, $T_{d}^{-1}$, and it fits to the universal growth law, $V_{b}=\left(\frac{1}{2}\right)V_{i}•2^{\frac{C+D}{T_{d}}}$.

We found that the mother cell length, width, and volume all decreased in longer channels (Figures 4A–C). The decrease was more pronounced in cell length and volume (17 and 33%) than that in cell width (7% change). This contrasts earlier measurements in Figure 3, where the cell width showed a larger variation while the length remained approximately constant. The decrease in cell length and volume was in good approximation proportional to channel length (*R* = −0.992 for both cases), while the decrease of cell width showed lower correlations (*R* = −0.832). The doubling time also increased linearly as a function of channel length (Figure 4D). In 50 μm long channels, the doubling time was 44% longer than in 15 μm long channels. The increase in doubling time and decrease in cell volumes were, in good approximation (*R* = 0.999), related by the universal growth law (Figure 4E). The fit parameters, which determine the cell volume at initiation per replication origin (*V*_*i*_), and the sum of C and D periods (Cooper and Helmstetter, 1968), matched those found from measurements where the channel width was varied (cf. Figures 3E, 4E). The consistency of these two datasets indicates that growth limitation likely had the same origin in the measurements where channel width and length were varied.

### Mechanical impediments to cell growth in 1D cultures

In the above measurements, the doubling time increased linearly as the function of channel length. We hypothesized that if the channel length increased even further then the doubling time of the mother cell would show a non-linear increase and perhaps cell growth would completely stop as the nutrient levels deplete at the end of the channel. To test if doubling time of mother cells as a function of channel length would deviate from linear at longer channel lengths, we fabricated a new batch of microfluidic chips with channel lengths of 20, 50, 100, 150, and 200 μm.

Unexpectedly, during the initial passivation step of the channel walls with bovine serum albumin (BSA), which preceded cell loading, we observed an accumulation of this protein to the channel ends in 100 μm and longer channels in phase contrast images. The effect was completely missing in channels 50 μm and shorter on the same chip even for long incubation times (>12 h). When the same M9 growth medium was used without BSA, no material accumulated to the ends of any channel. Aggregation of BSA was so strong that it completely excluded cells from the channel ends. Further investigation showed that the effect was not specific to BSA because if the channels were left with LB medium that did not contain any BSA, some material still accumulated to the channel ends. Similar effects have been observed before and were explained by water diffusing into PDMS (Randall and Doyle, 2005). The effect depends sensitively on channel length as will be discussed in more details later. Although BSA prevented cells from occupying the ends of 100 μm and longer channels, cells could also be cultivated without BSA. In this case, there was some aggregation of cells near the entrances of the channels. The omission of BSA thus increased sticking of cells to the channel walls, as expected. However, the growth rate of cells in 20–50 μm long channels without BSA passivation were not different from these with BSA (Supplementary Figure 4).

**Figure 5:**
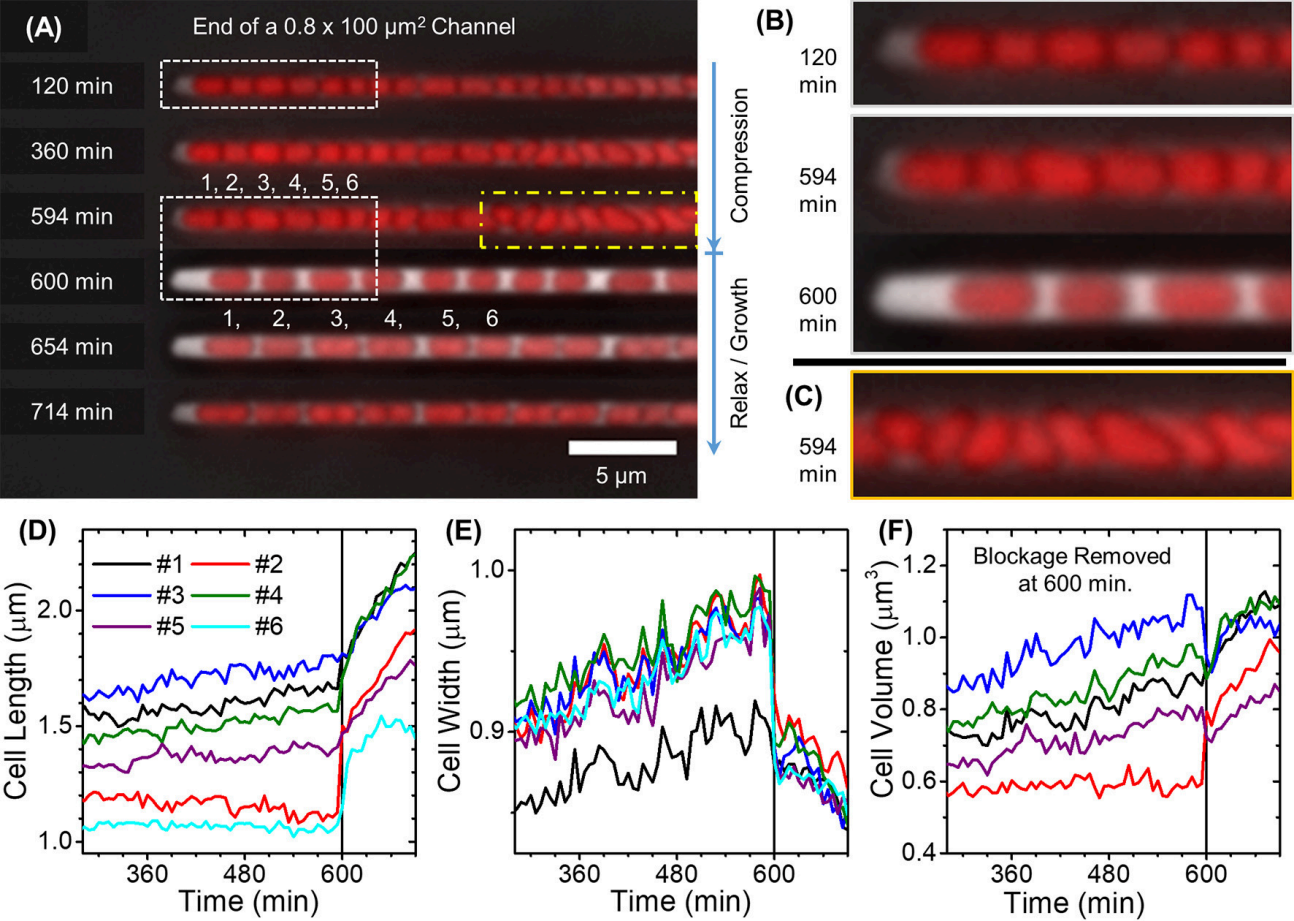
Cell growth in 100 μm long channels. **(A)** Composite time-lapse images of cells growing in a 100 μm long and 0.8 μm wide channel without BSA passivation. Pressure builds up in the channel until the frame at 594 min. Pressure release occurs between 594 and 600 min after which cell growth and division resume. Individual cells are labeled with numbers 1 to 6. **(B,C)** Boxed regions in **(A)** are magnified. **(D–F)** Cell lengths, widths and volumes as a function of time for the first six cells in the channel as labeled on **(A)**. Solid vertical lines show time when the pressure release occurred.

The growth of colonies in 100 μm and longer channels differed significantly from those in shorter channels (≤50 μm). These differences were present irrespective of the presence or absence of BSA in the growth medium. Attachments of cells to channel walls combined with their continued growth lead to considerable pressure buildup, which was evidenced by the widening of the channel in phase contrast images (Figures 5A–C). In deformed regions of the channel, the cells grow in multiple rows or were tilted relative to the direction of the channel. Based on our earlier studies (Yang et al., 2015), stress analysis of such channels has shown that pressures in the 0.2 MPa range are needed to widen channels at their midplane by few hundred nanometers. Similar or even larger pressures must have been present in the broadened regions of the channel. From time to time the pressure buildup resulted in a sudden release of cells from the channels as the force resulting from cell growth exceeded some critical value needed to break adhesive contacts between cells and channel walls (Figure 5A). However, in some channels, these contacts were likely stronger or differently distributed, and no pressure release occurred during the 12 h observation period.

The pressure buildup had pronounced effects on cell growth and morphology (Figure 5C, Supplementary Videos 4, 5). As the pressure increased, cell elongation and division rates slowed down (Figure 5D), and the cells started to broaden (Figure 5E). For cells that grew tilted relative to the channel axes in the initial stages of pressure buildup, the broadening was not uniform along the cell length due to uneven pushing from other cells (Supplementary Videos 6, 7). The cell regions in contact with the other cells expanded less than the regions where cells were able to expand toward channel walls without these contacts. As a result, the cells acquired irregular pear-shaped morphologies that significantly differed from the regular rod shape (Figure 5C). Some mass growth, albeit at a much slower rate than in liquid cultures, still occurred in these conditions (Figure 5F). The mass growth in these conditions could be predominantly attributed to cell broadening rather than to elongation. Upon release of pressure, the cell shape immediately returned to the usual elongated rod-shaped morphology. This sudden change was accompanied by a decrease in cell width (≈7%) and increase of cell length (≈10%). Interestingly, cell elongation resumed within minutes after pressure was released (Figure 5D) even though the elongation rate had been almost zero during the 9 h compression period. Along with resumption of cell elongation, the cell width, after decreasing abruptly upon release of compression, decreased further. Over the course of 1.5 h, it approached its regular value (0.78 μm). The slowdown of cell growth also appeared in measurements in 100 μm and longer channels when BSA was used (Supplementary Figure 5, Supplementary Video 8). The slowdown was followed by a period of faster growth after each pressure release. However, adhesion of cells to the channel wall was clearly weaker in this case; consequently, smaller pressure buildups occurred. In these conditions, cell morphology remained regular, and broadening could not be detected. Altogether, in 100 μm and longer channels, mechanical hindrances were the growth limiting factor instead of nutrient depletion.

## Discussion

### Cell shape shows adaptations to channel geometry

Here we studied how confinement changes the growth of *E. coli* cells in microfluidic channels of different widths and lengths. In 15 and 20 μm long channels with widths of 0.9 and 1.0 μm, the growth rate and cell volume were comparable and even slightly (5–10%) exceeded the values from liquid cultures measurements. Although the confinement related effects to the growth rate in these conditions were negligible, cell morphology was affected. Cells were significantly longer and narrower than in liquid cultures. What factors could have led to the change of cell morphology? In the narrowest channels with widths of 0.7 μm, the cell width was physically limited by the channel walls. The same mechanical constraint could also have limited cell diameter in 0.8 μm wide channels. However, in 0.9 and 1.0 μm wide channels the cells were not squeezed by channel walls, yet they still maintained a higher length to width ratio compared to liquid culture cells (2.7 vs. 2.1, respectively). Even higher ratios (>3.4) can be inferred from previous measurements by another group using mother machine devices at similar growth conditions (Taheri-Araghi et al., 2015). We hypothesize that cell shape in these channels is narrower and longer because of contacts to channel walls. Although the cell adhesion to channel walls was weak, especially to BSA passivated channel walls, these contacts may have had an effect on peptidoglycan synthesizing machinery. Consistent with this idea, the length to diameter ratio of cells was higher when channel walls were not passivated by BSA compared to that when they were (Supplementary Figure 4). By a common view, peptidoglycan synthesis activity is controlled via MreB and FtsZ polymer scaffolds, which reside in the cytosolic side of the inner membrane (Typas et al., 2012). The data presented here point to the possibility that stresses in the outer envelope may also modulate peptidoglycan synthesis activities directly without being transmitted via MreB and FtsZ scaffolds.

### Is mass transport limiting cell growth in channels?

Our measurements showed universal growth law-like dependence between cell volume at birth and growth rate. We interpreted this dependence as arising from nutrient limiting conditions at the channel. We hypothesized that adsorption of nutrients by cells growing between the mother cell and the channel entrance lead to depletion of nutrients at the location of the mother cell. This effect has been also referred to as nutrient shielding (Lavrentovich et al., 2013). Taking the simple geometry of channels and growth of bacteria in single rows without gaps, the nutrient shielding effects in these conditions can be quantitatively analyzed using 1D reaction-diffusion equations. Separate equations can be written for each chemical compound present in the growth medium. Here, we assume that there is just one component in the medium that is growth limiting. Denoting its concentration by *c*, its diffusion coefficient by *D*, its absorption coefficient per unit cell surface area by *k*_*abs*_, channel width by *W* and height by *H*, and cell radius by *R*_*c*_ a reaction-diffusion equation as a function of distance *x* from the channel entrance for this component can be written as:

(2) $$\left(W\;\cdot \;H-\pi R_{c}^{2}\right)\;\cdot \;D\frac{d^{2}c}{\;dx^{2}}=2\pi R_{c}k_{abs}\;\cdot \;c$$

Here we assumed that the uptake of this nutrient component is a first order process that is far from saturation. If the uptake were saturated (kinetically limited) then the corresponding compound would not be a growth limiting factor. The solution to this equation assuming non-adsorbing boundary condition at channel end is:

(3) $$c\left(x\right)=\frac{c_{0}\cosh\left(\frac{x-L}{\lambda }\right)\;}{\cosh\left(\frac{L}{\lambda }\right)}\;\lambda =\sqrt{\frac{\left(W\;\cdot \;H-\pi R_{c}^{2}\right)\;\cdot \;D}{2\pi R_{c}\;\cdot \;k_{abs}}}$$

Here *L* is length of the channel and *c*_0_ is concentration of this compound at the channel entrance. λ defines a characteristic length scale, which is referred to as the nutrient screening length (Lavrentovich et al., 2013). The screening length depends on the cross-sectional area of the channel, being longer for wider channels. We next assume that growth rate (inverse of doubling time), which is controlled by this compound, follows Michelis-Menten type of kinetics (Shehata and Marr, 1971):

(4) $$T_{d}^{-1}\left(x\right)=\frac{T_{d,min}^{-1}\;}{1+\frac{K}{c(x)}}$$

Here *T*_*d*_ is doubling time of cells at position *x* from the channel entrance, *T*_*d,min*_ is the doubling time in conditions where the compound is not growth limiting and *K* determines the concentration above which nutrient uptake rate starts to saturate. In dilute liquid cultures *T*_*d,liquid*_ = *T*_*d,min*_(1+*K*/*c*_0_). Using the latter expression and the solution for the reaction-diffusion equation for *c(x*) yields:

(5) $$T_{d}\left(x\right)=T_{d,min}+\left(T_{d,liquid}-T_{d,min}\right)\cosh\left(\frac{L}{\lambda }\right)/cosh\left(\frac{x-L}{\lambda }\right)$$

Our data concerns mother cells. The doubling time of the mother cell *T*_*d*_ then becomes:

(6) $$T_{d}=T_{d,min}+\left(T_{d,liquid}-T_{d,min}\right)\cosh\left(\frac{L}{\lambda }\right)$$

Here we assumed the position of the mother cell to be *x* ≈ *L*. The formula predicts that the doubling time in short channels *L* ≪ λ is independent of channel length and equals the doubling time in liquid cultures. In channels *L* ≈ λ it increases as $T_{d}\;\sim L^{2}$ and in channels *L* ≫ λ the increase is exponential *T*_*d*_ ~ *exp*(*L*/λ). Altogether equation 5 thus predicts a distinctly non-linear relationship between doubling time and channel length. In contrast to this prediction, the data in Figure 4D is in good approximation linear (Supplementary Figure 6). The discrepancy between the model and the data also appears when one compares dependence of the doubling time on channel width. The model predicts a smaller variation of doubling time as a function of channel width, in particular for 20 μm long channel (Supplementary Figure 6). It is possible that model treats too simplistically the relationships between concentration of growth-limiting compound and growth rate. For example, some deviations from a single Michelis-Menten type relationship for growth rates were observed in glucose limiting conditions (Shehata and Marr, 1971). However, the underlying relationship between doubling time and channel length should still be distinctly non-linear and as such not consistent with the experimental data. Moreover, there is no obvious compound in our growth medium that can be growth limiting. Concentrations of all media components at channel opening are in the millimolar range and as such exceed several orders of magnitude the growth-limiting concentrations. For example, concentration of glucose in the channel opening is 22 mM. This is about 10^4^ higher than its growth-limiting concentration of about 1 μM (Shehata and Marr, 1971). The uptake of glucose by cells is thus completely kinetically limited in the vicinity of channel opening. One can solve the reaction-diffusion equation similar to equation 1 also in kinetically limited regime. The solution shows that the concentration of such nutrients decreases quadratically from the channel entrance as:

(7) $$c=c_{0}-c\left(L\right)=\;L^{2}\frac{I_{\max}}{2\left(WH-\pi R_{c}^{2}\right)L_{c}D}$$

Here *I*_*max*_ is kinetically limited nutrient uptake rate per cell per second, and *L*_*c*_ is the average cell length. For glucose *I*_*max*_ = 2·10^5^ molecules per cell per second (Natarajan and Srienc, 1999) and *D* = 700 μm^2^/s (Longsworth, 1953) yields concentration change of 2.7 mM (~10%) at the end of 50 μm long channel that is completely filled with bacteria. Resulting 19 mM concentration of glucose in channel end corresponds still to highly saturating level. The same arguments also apply to the casamino acids, which are present in mM concentrations too. Moreover, their depletion with exception of leucine, which is essential for the strain, would not significantly alter the doubling times. If depletion of leucine would have occurred then this would have led to increase in doubling times approximately given by equation 5. Such dependence, however, would not be consistent with the experimentally observed linear dependence in Figure 4D as already argued.

The previous analysis assumed mass transport of nutrients to channels is solely via diffusion. However, accumulation of BSA to the ends of 100 μm and longer channels demonstrates that there is significant influx of water into these dead-end channels because of evaporation/diffusion water into PDMS (Randall and Doyle, 2005). This influx was significant even after channels were filled with water for a 12-h period. The steady uptake rate of water to PDMS has been estimated to be *J* = 7·10^−6^ kg/(m^2^s) (Randall and Doyle, 2005). This update rate explains why BSA accumulates in 100 μm long channels but not in 50 μm long channels. The steady state concentration profile for the BSA in empty channels can be found from equation that balances its diffusive and convection fluxes:

(8) $$W\;\cdot \;H\;\cdot \;D_{BSA}\frac{dc_{BSA}}{\;dx}=\frac{J}{\rho }\left(2H+W\right)\;\cdot \;(L-x)\;\cdot \;c_{BSA}(x)$$

Here ρ is density of water. The solution to this equation is:

(9) $$\begin{array}{l} c_{BSA}\left(x\right)=c_{0,BSA}\exp\left(\frac{L^{2}}{2\sigma^{2}}\right)\exp\left(-\frac{{\left(L-x\right)}^{2}}{2\sigma^{2}}\right)\;\sigma \\ \;=\sqrt{\frac{\rho \;\cdot \;D_{BSA}}{J}\frac{WH}{2H+W}} \end{array}$$

Here σ is a characteristic length scale for accumulation, which depends on cross-sectional parameters of the channel but not on its length. For BSA, which diffusion coefficient is 70 μm^2^/s, σ = 57 μ*m* can be estimated for 0.9 μm wide channels. Increase in BSA concentration in channel end is 1.5 times in 50 μm, 5 times in 100 μm and 500 times in 200 μm long channels. 500 times increase corresponds to almost complete precipitation of BSA as observed in our experiments.

While water permeation to PDMS has strong effect on the BSA distribution in the channel, Equation (8) predicts that it does not have significant effect on distribution of nutrients, signaling molecules and metabolic waste products in the channel. The reason is that diffusion coefficients of these small molecules are about an order of magnitude larger (e.g., *D* = 700 μm^2^/s for glucose) than for BSA. Even for 200 μm long empty channel one would expect the concentration of these molecules to be less than twice of that in the channel entrance. This increase does not likely have a significant effect on cell growth.

So far we did not consider adsorption of small molecules by channel walls. In current treatment, the adsorption would be completely analogous to adsorption of these molecules by cells. Consequently, adsorption by channel walls can be accounted by increasing *k*_*abs*_ in Equation (2) while the functional form of the equations remains the same. Accounting wall adsorption would therefore not lead to better agreement between experiment and model as inclusion of adsorption would not change functional dependence of doubling time on channel length.

Altogether our analysis shows that mass transport related limitations to cell growth in channels ≤ 100 μm are not significant in our experimental conditions despite our initial expectations. However, if some of the essential components in the media are present at less than saturating levels then the doubling times of the cells in the channel ends should become strongly affected. Flow of water into channels because of permeability of PDMS should not affect these conclusions because of fast diffusion of nutrients and waste molecules.

### Mechanical constraints to cell growth

Our experiments indicate that instead of nutrient limitations, the mechanical impediments set stronger constraints for bacterial growth in channels. In channels 100 μm and longer, friction forces caused cessation of cell elongation. In shorter channels friction could have also been the main growth limiting factor. In accordance with this assumption, friction leads to growth opposing force on a mother cell, *F*_*f*_, which increases proportionally to channel length *F*_*f*_ ~ *L*. Measurements of *E. coli* growth in agarose hydrogels have shown that forces opposing cell growth decrease elongation rate approximately linearly as the magnitude of the force increases (Tuson et al., 2012). For small forces, this would mean that increase in doubling time from the bulk value is proportional to the opposing force and thus on channel length Δ*T*_*d*_ = *T*_*d*_ − *T*_*d,liquid*_ ~ *F*_*f*_ ~ *L* in agreement with the experimental data (Figure 4D). Increased friction can also explain why in narrower channels doubling time increased (Figure 3D).

Although sticking of individual cells to channels walls was weak in channels longer than 50 μm, the cumulative effect of adhesion become strong enough to prevent cell elongation on the dead-end side of the channels. Interestingly, some residual mass growth still occurred in this situation that resulted in broadening of cells. Presumably due to this residual growth cells in some channels were able to overcome static friction forces and release the pressure that opposed their growth. During the release of pressure, the cells behaved similarly to a compressed elastic rod: their length increased (25%) and diameter decreased (7%). The length increase furthermore indicates that cells did synthesize new cell wall during the compression phase. Taken the previously estimated Young modulus in the range of 50–150 MPa for *E. coli* cell for the envelope thickness of 4 nm (Tuson et al., 2012) 25% of compression corresponds to force on the 0.8 μm wide cell of 0.1–0.4 microNewtons (μN). Here we assumed that turgor pressure, during and few minutes after the compression, was the same and that cell wall compression was elastic. The force of 0.1–0.4 μN then corresponds approximately to the stall force for peptidoglycan synthesis in *E. coli*. For comparison, the stalling force has been estimated to be 11 μN in fission yeast (Minc et al., 2009).

Strikingly, the cells were able to restore their growth rates very rapidly (less than 6 min) after more than 9 h of very limited growth during compression. Here the 6 min estimate corresponds to the frame rate of measurements, but actual lag phase could have been shorter. Very short lag time if any is in contrast with the time needed to restore growth in stationary phase cells upon entering fresh medium. In our measurements, this time has typically been in the range of 0.5–2.0 h depending on the length of time cells spent in stationary state. A period of approximately 30 min appears to be needed to restore transcriptional activity and assemble functional peptidoglycan synthesis complexes. Since the cell growth in channels resumed much faster, the cells must have maintained active peptidoglycan machinery throughout the compression period. The compressed cells in channels were clearly different from stationary phase cells as they had access to nutrients and were metabolically more active. Maintenance of enzymatic machinery, even when no elongation occurred, indicates that mechanical stress opposing cell growth alone is not able to suppress transcriptional peptidoglycan synthesis machinery the way the entry to stationary phase does.

### Implications to bacterial growth in microfluidic devices and in natural microenvironments

Our work brings out some differences in the growth of *E. coli* in confined, relative to unconfined, conditions in mother machine platform. We find that for mother machine platform the channels need to be in a rather narrow range of dimensions for not to display growth-limiting effects: for *E. coli* growing in M9 medium with glucose and casamino acids only 15 and 20 μm long and 0.9 and 1.0 μm wide channels did not display these effects. However, even in these channels cell morphology differed from the one in liquid cultures. The different aspect ratio that we observed is not likely significant factor for most experiments, but for those dealing with cell size control and cell wall synthesis, these effects need to be considered. To improve the mother machine design, one can use even shorter (<15 μm) channels. However, this comes with the drawback of losing cells from the channel in long-term experiments. On the other hand, our data and analysis indicate that growth limitation due to nutrient and waste diffusion is minimal even in long channels (>100 μm). The designs that increase mass transport to channels, such as done recently by diverting some fluid flows through the channels (Baltekin et al., 2017; Jennings, 2017) or using shallow side channels that surround the cells (Norman et al., 2013; Cabeen et al., 2017), is not likely to alleviate growth limitations in these channels.

The mechanical constraints resulting from cell adhesion may be a major limiting factor for bacterial growth not only in microfluidic channels but also in natural environments such as soil where small pores and channels with dimensions comparable to bacterial size are present (Ranjard and Richaume, 2001). These constraints may also limit bacterial growth in unconstrained colonies such as ones on agar plates. Although nutrient limitation has been considered as the main growth limiting factor for growth of bacterial cells in the interior of the colony (Jeanson et al., 2015), our results point out the possibility that mechanical constraints have an equally important role in limiting growth only to the outer layers of the colony.

## Author contributions

DY and JM contributed conception and design of the study. DY and SR fabricated the devices used in the study. DY, AJ, EB performed the measurements. DY analyzed the data. DY and JM wrote the manuscript. All authors contributed to manuscript revision, read and approved the submitted version.

### Conflict of interest statement

The authors declare that the research was conducted in the absence of any commercial or financial relationships that could be construed as a potential conflict of interest.
