# Supplementary material for: Analysis of Factors Limiting Bacterial Growth in PDMS Mother Machine Devices
Source: Front Microbiol. 2018 May 1;9:871. doi: 10.3389/fmicb.2018.00871 (PMC5938360; doi:10.3389/fmicb.2018.00871)
Supplement: Supplementary file 9 [file Presentation_1.pdf]

Supplementary Material

Mass transport and mechanical limitations to bacterial growth in one dimensional colonies

Da Yang, Anna D. Jennings, Evalynn Borrego, Scott T. Retterer, Jaan Männik*

*** Correspondence:** Jaan Männik: JMannik@utk.edu

1 Supplementary Videos

Videos S1, S2: Accompanying Figure 2--cells growing in 0.8 x 15 µm2 and 0.9 x 20 µm2 channels.

Video S3: Accompanying Supplementary Figure S2--Cells moving out from 0.7 um channel.

Video S4: Accompanying Figure 5--cells in 100 um long channel w/o BSA.
Video S5: A second example.

Videos S6, S7: Long channels with cells grew titled relative to channel axes in the initial stages of pressure buildup, the broadening was not uniform.

Video S8: Accompanying Supplementary Figure S5--cells in 100 um long channel with BSA.

2 Supplementary Figures

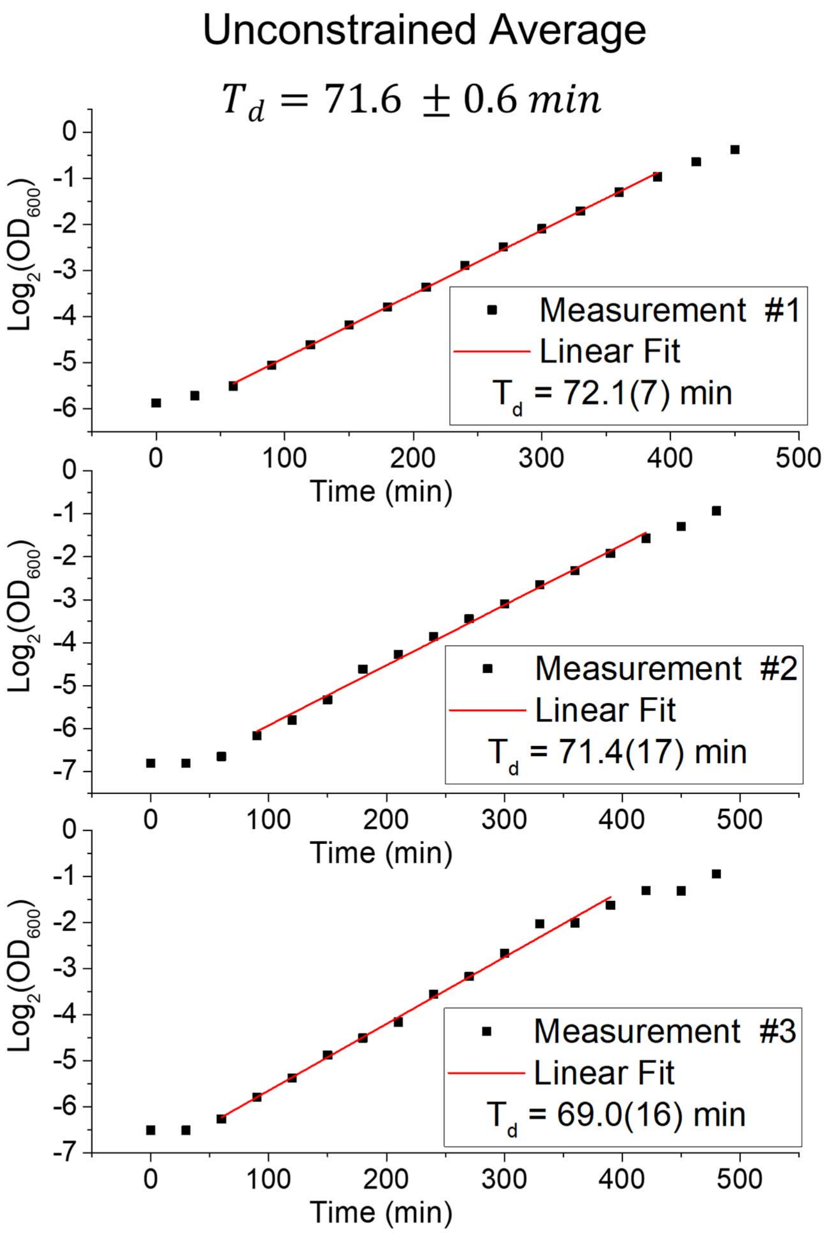

Supplementary Figure 1. Three independent measurements of OD_{600} vs time for AJ5 strain. Cells are growing at T= 28°C in M9 minimal medium with glucose and casamino acids (the same growth conditions as in microfluidic chip measurements). Solid lines represent linear fits from which doubling times are determined. The weighted average doubling time from three measurements is $T_d = 71.6 \pm 0.6\ min$ (s. e. m).

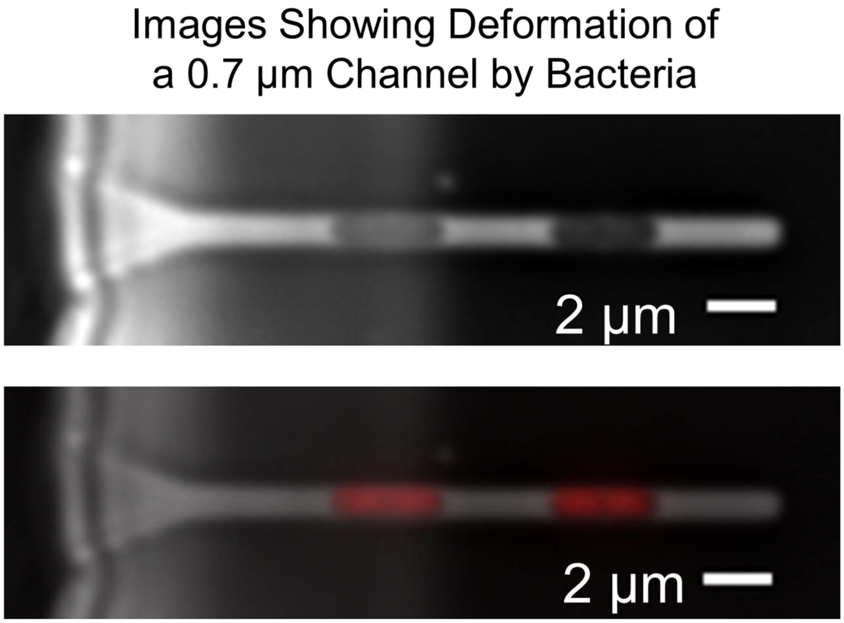

Supplementary Figure 2. Images of cells in 0.7 µm wide and 15 µm long channel. Top panel is a phase contrast image and bottom overlay of phase contrast and fluorescence images. Broadening of the channel around cells is visible. The broadening is less apparent in 0.7 µm wide channels that are completely filled with cells because in this case the channels are uniformly deformed. Note that analysis for Figure 3 in the main text includes only data from channels that are completely filled with cells unlike shown in this Figure. The whole movie of growth and division of these cells are presented in Supplementary Video S3. The Figure here corresponds to frame at 264 minutes in Supplementary Video S3.

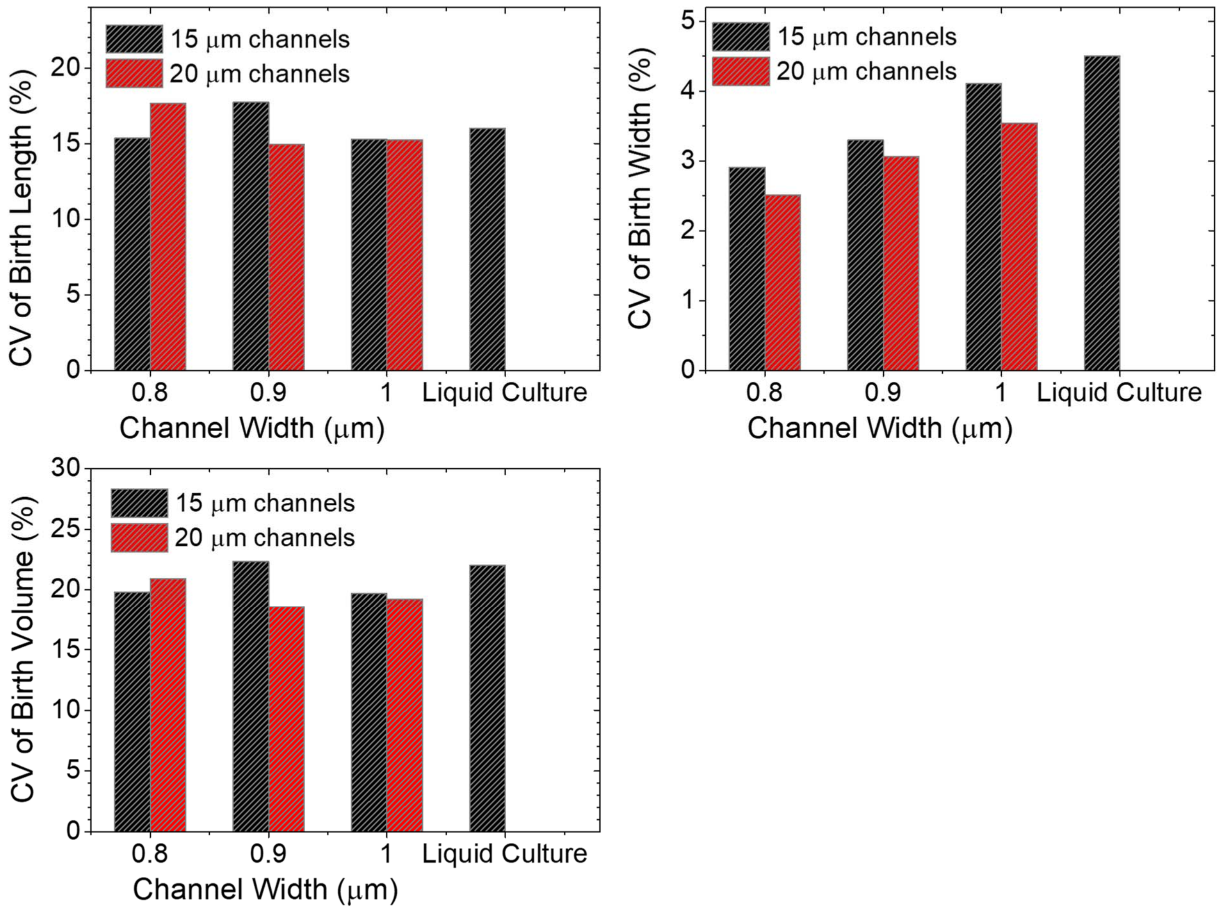

Supplementary Figure 3. Coefficient of variation (CV) for cell length, width and volume distributions. All the distributions have been measured at cell birth. Coefficient of variation here is a pooled standard deviation from three independent measurements divided by a pooled mean.

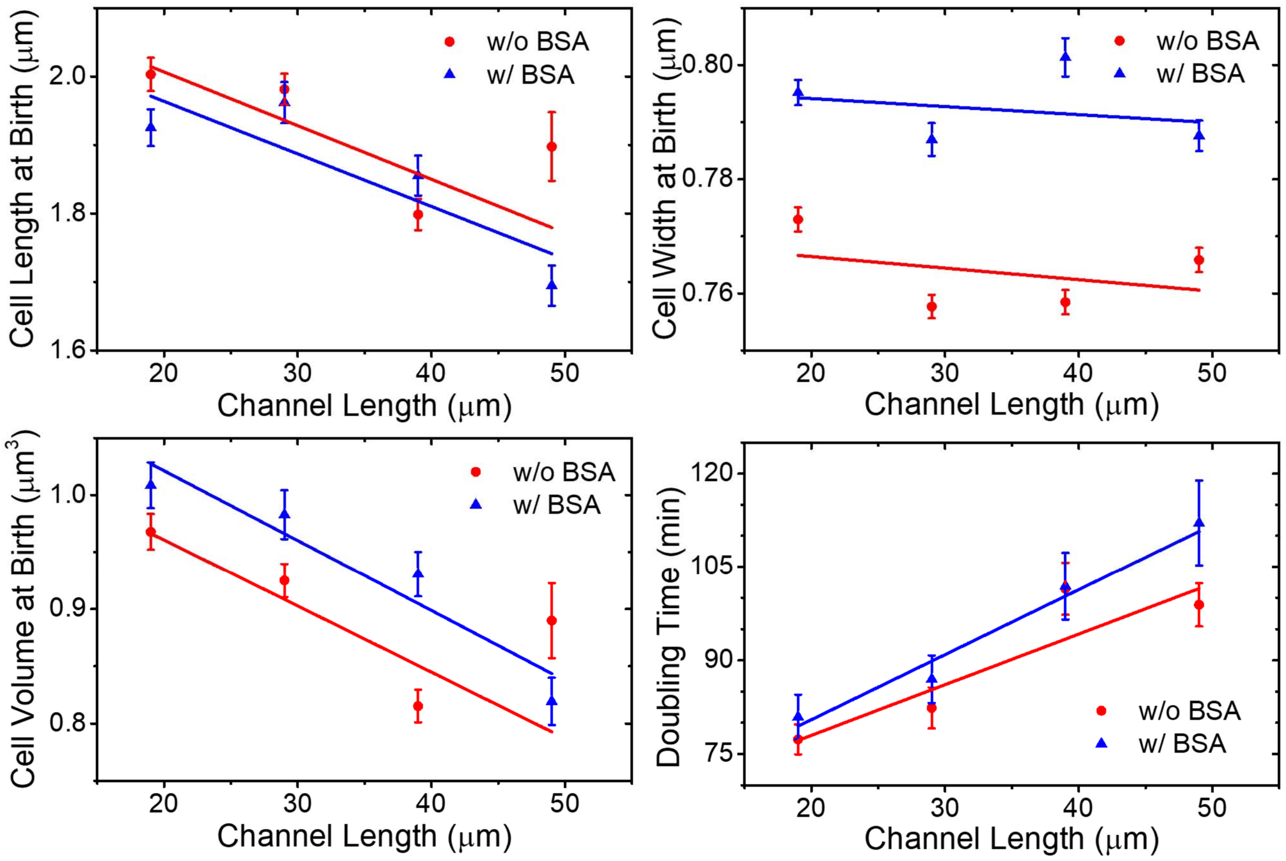

Supplementary Figure 4. Mother cell length, width, volume and doubling time as a function of channel length for channels with and without BSA passivation. All cell dimensions are determined at cell birth. All channels are 0.9 μm wide and 1.15 μm high. Note that the same channels and mother cells were used for the two measurements. The measurements without BSA were performed first and then the growth medium was switched to one that contains 0.1% BSA. 12 hours was waited before starting a new measurement to ensure proper BSA passivation and to allow cells to adjust to new growth conditions.

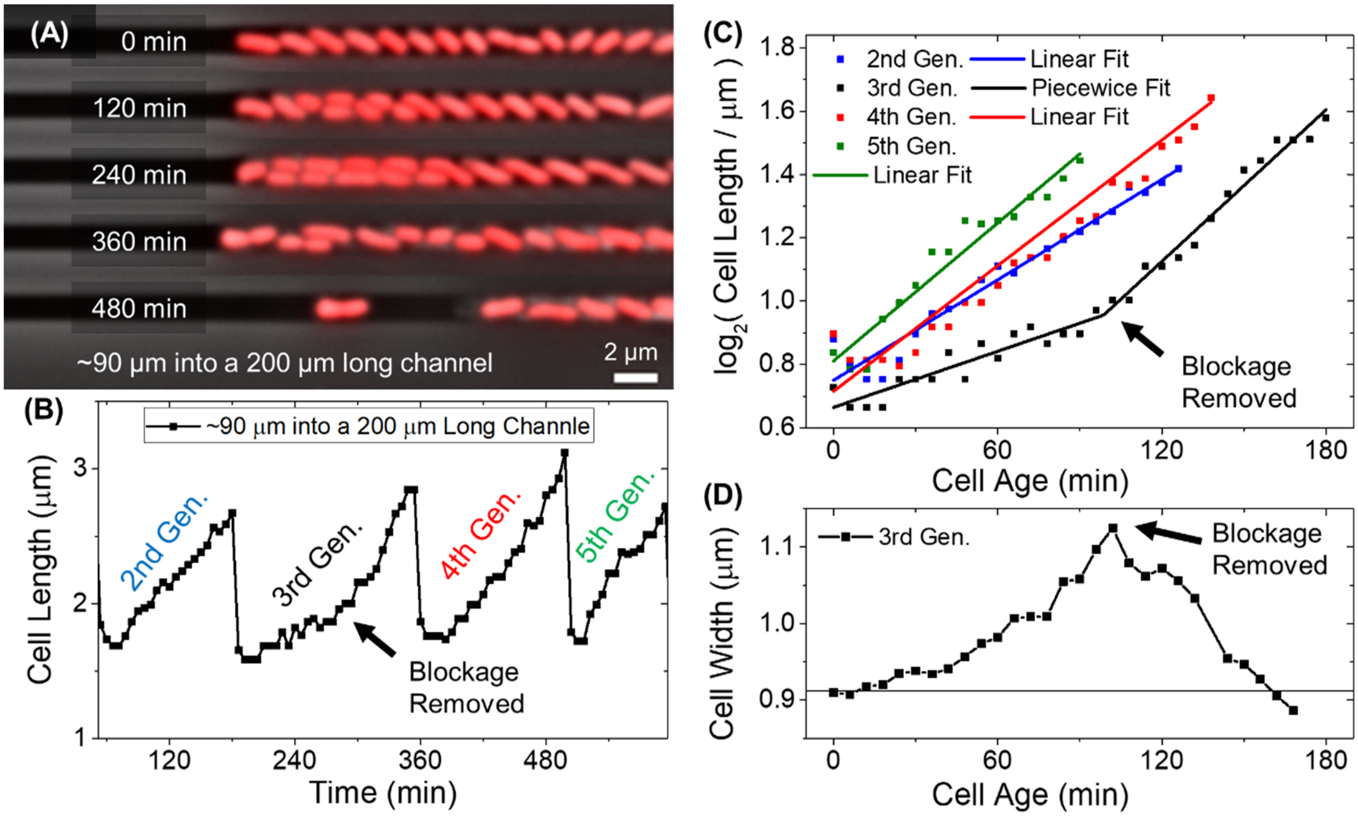

Supplementary Figure 5. Cell growth in 200 µm long channels with 0.1% BSA. (A) Composite time-lapse images of cells growing in a 200 µm long and 0.8 µm wide channel. The images have been captured at about 90 µm from the channel entrance. Cells do not fill the channel further than about 90 µm because of BSA accumulating to channel ends. BSA accumulation leads to darkening of channels in phase contrast images. In this measurement, pressure builds up in the channel until 300 minutes. (B) The elongation of the mother cell in this channel as a function of time. The elongation slows down as the pressure builds up in the channels and increases when the blockage to cell movement disappears. (C) Mother cell elongation in logarithmic scale plotted as function of cell age. (D) Width of the mother cell before and after the blockage disappears.

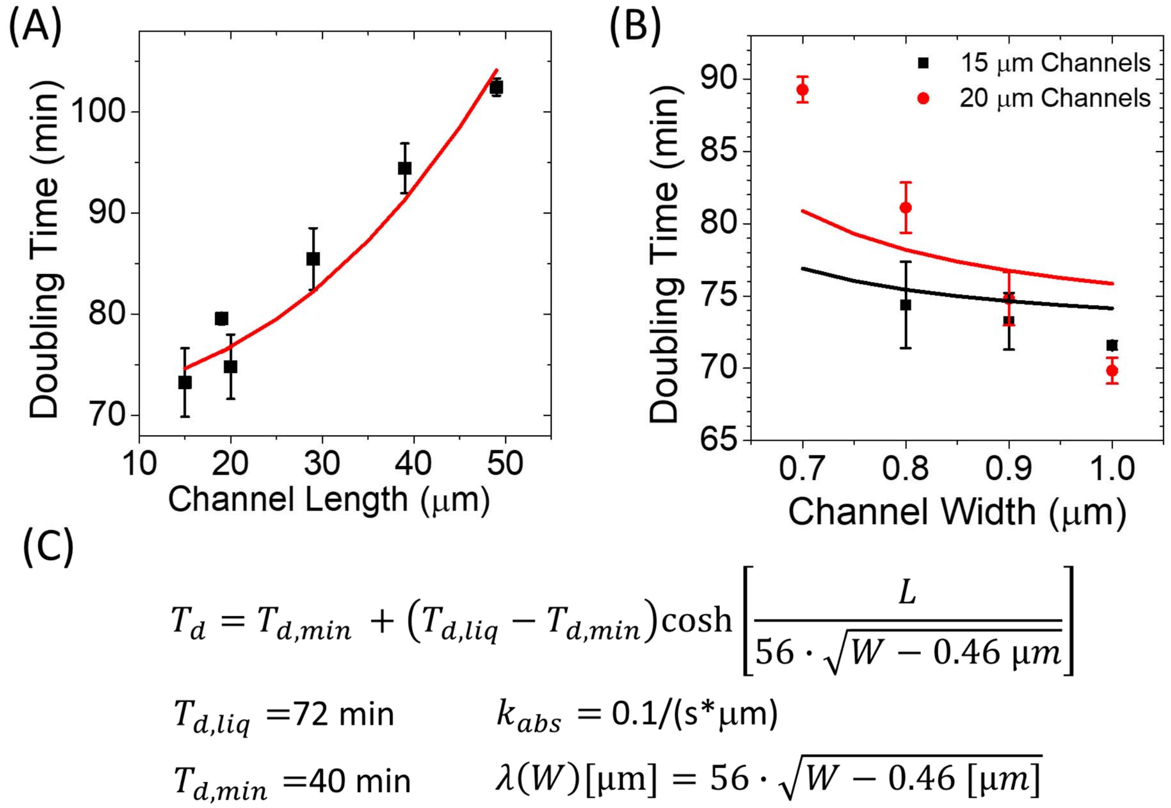

(C)

$$T_d = T_{d,min} + \left(T_{d,liq} - T_{d,min}\right)\cosh\left[\frac{L}{56 \cdot \sqrt{W - 0.46\ \mu m}}\right]$$

$T_{d,liq}$ =72 min $\qquad k_{abs} = 0.1/(\text{s}*\mu\text{m})$

$T_{d,min}$ =40 min $\qquad \lambda(W)[\mu\text{m}] = 56 \cdot \sqrt{W - 0.46\ [\mu m]}$

Supplementary Figure 6. Comparing modelling and experimental data on doubling times. (A) Doubling time as function channel length (the same data as in Figure 4D). All channels are 0.9 μm wide. Solid line is fitting with the Equation 5 from the main text. $T_{d,liquid}$ is taken as 72 min based on measurements of liquid cultures. The best agreement is reached as small as possible $T_{d,min}$. However, the shortest doubling time at T =28 °C of our *E. coli* strain cannot possibly be shorter than 40 min. The latter correspond to doubling time of this strain in LB medium at this temperature. Accordingly, $T_{d,min}$ is fixed at 40 min. Fitting yields nutrient screening length $\lambda_{fit} = 37$ μm. From the screening length absorption rate $k_{abs} = 0.1\ (s \cdot \mu\text{m})^{-1}$ can be calculated using Equation 2 of the main text assuming diffusion constant $\text{D} = 700\ \mu\text{m}^2/\text{s}$ (corresponding to glucose). (B) Using the estimated $k_{abs} = 0.1\ (s \cdot \mu\text{m})^{-1}$ dependence of doubling time on channel width is plotted against experimental data from Figure 3D in the main text. (C) Fitting function with the parameters used.
